# Neural Precursor Cell-Expressed Developmentally Downregulated Protein 4 (NEDD4)-Mediated Ubiquitination of Glutathione Peroxidase 4 (GPX4): A Key Pathway in High-Glucose-Induced Ferroptosis in Corpus Cavernosum Smooth Muscle Cells

**DOI:** 10.3390/biom14121552

**Published:** 2024-12-05

**Authors:** Wenchao Xu, Peng Hu, Jiaxin Wang, Hongyang Jiang, Tao Wang, Jihong Liu, Hao Li

**Affiliations:** 1 Department of Urology, Tongji Hospital, Tongji Medical College, Huazhong University of Science and Technology, Wuhan 430030, Chinajhliu@tjh.tjmu.edu.cn (J.L.); 2 Institute of Urology, Tongji Hospital, Tongji Medical College, Huazhong University of Science and Technology, Wuhan 430030, China

**Keywords:** erectile dysfunction, CCSMCs, GPX4, ferroptosis, high glucose, NEDD4

## Abstract

Pharmacological treatment of diabetes mellitus-induced erectile dysfunction (DMED) has become increasingly challenging due to the limited efficacy of phosphodiesterase type 5 inhibitors (PDE5i). As the global prevalence of DM continues, there is a critical need for novel therapeutic strategies to address DMED. In our previous studies, we found that Glutathione peroxidase 4 (GPX4), a ferroptosis inhibitor, can ameliorate DMED in diabetic rats. However, the specific role of GPX4 in corpus cavernosum smooth muscle cells (CCSMCs) and its regulatory mechanisms remain unclear. In this study, we established primary cultures of CCSMCs and systematically analyzed the role of GPX4 under high-glucose conditions. To further elucidate the upstream regulatory pathways of GPX4, we employed immunoprecipitation coupled with mass spectrometry (IP-MS) to identify potential interacting proteins. Additionally, co-immunoprecipitation (Co-IP) and cycloheximide (CHX) chase assays were conducted to explore the regulatory dynamics and post-translational stability of GPX4. Under high-glucose conditions, the expression of GPX4 in CCSMCs is significantly downregulated, leading to an increase in intracellular oxidative stress and heightened levels of ferroptosis, accompanied by dysfunction in smooth muscle cell relaxation. Furthermore, the CHX chase assay revealed that high glucose accelerates GPX4 protein degradation via the ubiquitin–proteasome pathway. Subsequent IP-MS identified NEDD4, an E3 ubiquitin ligase, as a potential interacting partner of GPX4. Further validation demonstrated that NEDD4 modulates the ubiquitination process of GPX4, thereby influencing its stability and expression. In conclusion, we identified NEDD4 as a key regulator of GPX4 stability through ubiquitin-mediated proteasomal degradation. These findings suggest potential therapeutic strategies targeting the NEDD4-GPX4 axis to alleviate DMED pathology.

## 1. Introduction

Erectile dysfunction (ED), a common andrologic disorder, affects up to 75% of individuals with diabetes mellitus (DM) [1]. The International Diabetes Federation predicts that the global diabetic population will increase by 25% by 2030 [2]. Notably, ED tends to manifest earlier and more severely in diabetic patients compared to the general population [3]. This highlights the urgent need for focused treatment and management strategies for DM-induced erectile dysfunction (DMED). While phosphodiesterase type 5 inhibitors (PDE5i) are the first-line treatment for ED, their efficacy is notably reduced in DMED patients, with only 44% showing a positive response [4]. Therefore, identifying novel and effective therapeutic targets for DMED is critical in advancing uro-andrological research and improving patient outcomes.

DM is a metabolic disorder characterized by chronic low-grade inflammation [5]. Studies have shown that inflammatory cytokines such as TNF-α and IL-6 are significantly elevated in patients with type 2 diabetes [6,7]. This pro-inflammatory environment may accelerate the dysfunction of pancreatic β-cells, ultimately leading to diabetic complications [8], including DMED. The pathophysiological mechanisms of DMED are marked by oxidative stress, increased ferroptosis, and impaired smooth muscle cell function [9,10]. Recent studies have highlighted the pivotal role of chronic inflammation in the onset and progression of DMED [11]. Inflammation exacerbates the condition by promoting the production of reactive oxygen species (ROS) and cellular damage. Furthermore, ferroptosis, a form of regulated cell death driven by iron-dependent lipid peroxidation, has been closely linked to inflammatory responses [12,13]. Research indicates that ferroptosis not only aggravates cellular injury but also releases damage-associated molecular patterns (DAMPs), which further activate inflammatory pathways, creating a vicious cycle [14]. Furthermore, corpus cavernosum smooth muscle cells (CCSMCs) play a critical role in penile erection. Upon sexual stimulation, nitric oxide (NO) is locally released in the penis, leading to the relaxation of CCSMCs and subsequent penile engorgement and erection [3]. Conversely, the RhoA/ROCK signaling pathway negatively regulates smooth muscle relaxation by increasing calcium sensitivity and intracellular calcium levels, promoting smooth muscle contraction [15]. Studies have shown that hyperglycemia activates the RhoA/ROCK pathway in CCSMCs, impairing their function and ultimately contributing to the development of DMED [16,17].

Iron imbalance, both deficiency and overload, is implicated in various diabetic complications, contributing to cellular dysfunction through oxidative damage [18]. Iron metabolism and lipid peroxidation are closely linked to ferroptosis, which is characterized by metabolic abnormalities that generate lipid peroxides, disrupting redox balance and leading to cell death [19]. Recent studies have demonstrated that ferroptosis is induced in diabetic conditions, particularly in vascular endothelial cells and smooth muscle cells, which play crucial roles in penile erection [20]. This highlights the potential involvement of ferroptosis in the pathogenesis of DMED, suggesting that ferroptosis in CCSMCs under high-glucose (HG) conditions may contribute significantly to the development of this disorder. Glutathione peroxidase 4 (GPX4) plays a vital role in regulating ferroptosis by reducing lipid peroxides, maintaining redox balance, and protecting cells from oxidative damage [21]. This crucial enzyme in the glutathione metabolic pathway is indispensable for ferroptosis inhibition [22]. In our previous studies using in vivo DMED rat models, we demonstrated that GPX4 improves erectile function by modulating ferroptosis, particularly affecting the number and function of CCSMCs in the penis [20]. However, the precise role of GPX4 in CCSMCs and the mechanisms regulating its expression under high-glucose conditions remain to be fully elucidated.

The ubiquitin–proteasome system (UPS) orchestrates the controlled degradation of nearly 80% of intracellular proteins, playing a critical role in maintaining cellular homeostasis [23,24]. Disruption of UPS function can lead to pathological conditions, including cancer, metabolic disorders, and neurodegenerative diseases [25,26]. The ubiquitination process involves a cascade of enzymatic reactions carried out by E1, E2, and E3 enzymes, with the E3 ubiquitin ligase being responsible for substrate recognition and the transfer of ubiquitin (Ub) to target proteins [27]. E3 ligases are classified into several families, including RING, HECT (homology to E6-AP carboxy terminus), and RBR types [28]. The neural precursor cell-expressed developmentally downregulated protein 4 (NEDD4), one of the most studied HECT E3 ligases, has been shown to play critical roles in both physiology and disease [29,30]. Recent research highlights ubiquitination, particularly mediated by NEDD4, as a key regulator of cellular ferroptosis [31,]. However, the specific role of NEDD4 in regulating GPX4 ubiquitination in CCSMCs remains unexplored.

Therefore, we hypothesize that GPX4-mediated ferroptosis may play a key role in regulating CCSMC function, with upregulation of GPX4 potentially reducing ferroptosis and oxidative stress, while enhancing cell viability and improving smooth muscle cell relaxation function. To further investigate the underlying regulatory mechanisms, we will employ immunoprecipitation coupled with mass spectrometry (IP-MS) to identify potential GPX4-interacting proteins.

## 2. Materials and Methods

### 2.1. Animal and Experimental Designs

This study, which included both in vivo experiments and CCSMC isolation, was approved by the Institutional Research Ethics Committee of Tongji Hospital (IACUC number: TJH-202106003). For in vivo experiments, sixteen eight-week-old male Sprague-Dawley (SD) rats were obtained from the Experimental Animal Center of Tongji Hospital, Tongji Medical College, Huazhong University of Science and Technology. As previously described, a DMED model was established and validated [20]. Briefly, ten rats received a single intraperitoneal injection of 1% streptozotocin solution (65 mg/kg, S0130, Sigma-Aldrich, St. Louis, MO, USA), while six control rats were injected with citrate buffer solution (0.1 mol/L, pH 4.5, C0759, and C3674, Sigma-Aldrich). To confirm DM, fasting blood glucose levels were measured 72 h post-injection, and rats with glucose levels above 16.7 mmol/L were classified as diabetic. Eight weeks after the DM model was induced, eight diabetic rats survived. Apomorphine testing was then conducted to identify rats with DMED, with those showing negative responses being classified as DMED. In the end, a total of six DMED rats were determined.

### 2.2. Cell Culture

For the isolation and culture of CCSMCs, we followed a standardized procedure in our laboratory to extract these cells from rat penile tissue (IACUC number: TJH-202106003) [32]. The CCSMCs were then cultured in DMEM (low-glucose) medium (PYG0106, Boster, Wuhan, China) supplemented with 10% fetal bovine serum (FBS, 10099-141, Gibco, Brisbane, Australia) and incubated at 37 °C with 5% CO_2_. To ensure a pure population of CCSMCs, a differential adhesion method was applied. Cell identification was confirmed using immunofluorescence labeling with α-SMA and Desmin (Table S1), as well as cytomorphological analysis. For subsequent experiments, cells from the third or fourth passage were used. Human embryonic kidney 293T (HEK-293T) cells (ATCC, CRL-3216) were cultured in DMEM (high-glucose) medium (PYG0073, Boster) with 10% FBS under the same controlled conditions of 37 °C, 5% CO_2_, and 95% humidity.

### 2.3. Cell Counting Kit-8 (CCK-8) Assay

To assess the impact of specific treatments on CCSMC viability, 10^4^ cells per well were seeded into 96-well plates and cultured for 24 h. Prior to treatment, CCSMCs were incubated in serum-free DMEM for 12 h. After treatment for the specified time periods, cell viability was measured using the CCK-8 assay. In brief, 10 μL of CCK-8 reagent (40203ES80, Yeasen, Shanghai, China) was added to each well, followed by a 1 h incubation in the cell incubator. Absorbance was then measured at 450 nm using a microplate reader (Thermo Scientific, New York, NY, USA).

### 2.4. Lentivirus Construction and Transfection

Lentivirus construction was based on a second-generation lentiviral system. Briefly, HEK-293T cells were seeded into 10 cm plates and allowed to grow until reaching 70–80% confluence, at which point they were prepared for transfection. Two hours prior to transfection, the cells were incubated in a serum-free DMEM (high-glucose) medium. A plasmid-transfection reagent mixture was then added to the cells, followed by a 6–8 h incubation at 37 °C. After this, the medium was replaced with a complete culture medium. The cell supernatant was collected and centrifuged at 4000× *g* for 10 min at 4 °C. Lentivirus-rich supernatants were filtered using a 0.45 μm syringe filter. The lentivirus was concentrated, harvested, and stored at −80 °C. A lentivirus lacking the transgene (ovNC) was produced as a negative control using the same procedure. For CCSMC transfection, CCSMCs were seeded into 6-well plates and allowed to grow until reaching 40–50% confluence. A lentivirus–transfection reagent mixture was then added to the cells at a multiplicity of infection (MOI) of 100, followed by a 24 h incubation. The medium was then replaced with a complete culture medium, and after an additional 48 h of culture, cells were selected using a culture medium containing 1:5000 puromycin.

### 2.5. Plasmid Construction and Transfection

After total RNA extraction, cDNA was synthesized using the HiScript II 1st Strand cDNA Synthesis Kit (R211, Vazyme). The full-length GPX4 coding sequence was amplified and cloned into pCMV-C-FLAG vectors. DNA fragments encoding NEDD4 and ubiquitin (Ub) were inserted into pCMV-C-MYC vectors. All DNA fragments were cloned into empty backbones using infusion cloning with the ClonExpress II One Step Cloning Kit (C112, Vazyme, Nanjing, China). The various plasmids were then transfected into cells for 24–48 h using Lipofectamine 3000 Reagent, following the manufacturer’s instructions (L3000075, Invitrogen, New York, NY, USA).

### 2.6. SiRNA-Mediated Knockdown

CCSMCs were cultured and seeded in 6-well plates at a density of 70–80%. The culture medium was replaced with Opti-MEM (31985062, Gibco) prior to transfection. For each well, 5 μL of siRNA was transfected using 5 μL of Lipofectamine Lipo2000 (11668019, Invitrogen). After transfection, the medium was replaced with the indicated medium containing 10% FBS, and the cells were incubated for 24 h. The siRNA sequence information can be found in Table S2.

### 2.7. Immunofluorescence Staining

CCSMCs were seeded on coverslips and fixed with 4% paraformaldehyde (PFA) for 30 min, followed by permeabilization with 0.5% Triton X-100 in PBS for 20 min at room temperature. After permeabilization, the cells were blocked using a blocking buffer (5% BSA in PBS) for 30 min. They were then incubated overnight at 4 °C with the corresponding primary antibody including anti-α-SMA, Desmin or GPX4 (Table S1). The following day, the coverslips were washed three times with PBS for 10 min each, and then incubated with Alexa Fluor-conjugated secondary antibodies (Table S1) for 1 h at 37 °C. After three more PBS washes, the nuclei were stained with DAPI.

### 2.8. Western Blotting

Protein expression levels were assessed via SDS-PAGE and followed by Western blotting. Primary antibodies including anti-GPX4, SLC7A11, ACSL4, LPCAT3, ALOX15, ALOX12, β-Actin, NOX1, NOX2, NOX4, ROCK1, ROCK2, RhoA, Ub, FLAG, NEDD4, and MYC (Table S1) were applied and incubated overnight at 4 °C to detect target proteins. Additionally, 4-HNE, a marker of lipid peroxidation, forms stable covalent adducts with proteins under oxidative stress. These 4-HNE-protein adducts can be detected using specific anti-4-HNE antibodies (Table S1) through Western blotting. After membrane washing, the appropriate secondary antibodies (Table S1) were applied for 1 h at room temperature. Protein bands were visualized using a Western ECL substrate, and band intensity was quantified using ImageJ software (v2.1.0) for densitometric analysis.

### 2.9. Quantitative Real-Time Polymerase Chain Reaction (qPCR)

Total RNA was extracted from penile tissues or CCSMCs using TRIzol reagent, following the manufacturer’s instructions (9108, Takara, Kusatsu, Japan). RNA concentration was determined by measuring optical density at 260 nm with a spectrophotometer (Thermo Scientific). cDNA was synthesized from the purified RNA using a cDNA synthesis kit (11120ES, Yeasen). Gene expression levels were quantified using the SYBR Green method (11202ES, Yeasen) and analyzed using the relative quantification approach. The primer sequence information can be found in Table S3.

### 2.10. Oxidative Stress Levels, Ca^2+^, and Iron Contents Detection

Oxidative stress levels in CCSMCs were assessed according to the manufacturer’s protocols using malondialdehyde (MDA), superoxide dismutase (SOD), glutathione (GSH), and glutathione peroxidase (GSH-Px) assay kits (A003-1, A001-1, A061-1, and A005-1, Nanjing Jiancheng, Nanjing, China, respectively). All the assay kits are based on colorimetric reactions and were detected using a microplate reader (Thermo Scientific). Total protein concentrations were used to normalize the oxidative stress markers. Reactive oxygen species (ROS) levels were measured using a Dihydroethidium (DHE) fluorescence probe technique (S0063, Beyotime, Shanghai, China), following the kit’s instructions. Briefly, cells were plated 24 h prior to the assay. The culture medium was aspirated, and the cells were washed three times with a serum-free culture medium. Serum-free culture medium containing 10 µmol/L of DHE probe was then added to each well, and the cells were incubated in the dark for 30 min. Fluorescence microscopy was used to observe, photograph, and record the results immediately afterward. Additionally, Ca^2+^ and iron levels in CCSMCs were quantified using assay kits (S1063S, Beyotime and A039-2, Nanjing Jiancheng, respectively), with results normalized to total protein concentrations.

### 2.11. Cycloheximide (CHX) Chase Assay

The protein stability of GPX4 was evaluated using the CHX chase assay. Following high-glucose treatment, CCSMCs were incubated with 100 μM CHX (HY-12320, MedChemExpress, Shanghai, China) to inhibit the protein synthesis translocation step. Cells were collected at designated time points, and GPX4 protein levels were analyzed by Western blotting. To investigate the specific degradation system of GPX4 protein, CCSMCs were treated with high glucose, followed by a 4 h intervention with either MG132 (a proteasome inhibitor, 10 μM, HY-13259, MedChemExpress) or 3-MA (a lysosome inhibitor, 10 mM, HY-19312, MedChemExpress) before sample collection. The cell samples were then collected for Western blot analysis.

### 2.12. Co-Immunoprecipitation (Co-IP) Assay

Penile tissues or CCSMCs were collected and lysed in RIPA buffer containing protease and phosphatase inhibitor cocktails. The cell lysates were prepared for immunoprecipitation, with 5% of the lysate reserved as an input control. The supernatant was transferred to fresh tubes and incubated overnight with anti-FLAG or IgG antibodies (Table S1) on a rotator at 4 °C. Afterward, the samples were incubated with protein A/G magnetic beads at 25 °C for 2 h. The beads were then collected and washed, and the bound proteins were eluted by heating at 95 °C for 10 min in 50 μL of SDS loading buffer. The immunoprecipitates were separated by SDS-PAGE and analyzed by Western blotting.

### 2.13. Immunoprecipitation Coupled with Mass Spectrometry (IP-MS)

CCSMCs were cultured and transfected with the GPX4-FLAG plasmid under high-glucose conditions, in the presence of the proteasome inhibitor MG132 (10 μM) prior to harvesting. The cell lysates were centrifuged at 12,000× *g* for 10 min, and the supernatant was incubated overnight at 4 °C with FLAG antibody (Table S1) on a shaker. Following this, the mixture was incubated with pre-washed protein A/G magnetic beads at 25 °C for 4 h. The beads were collected and washed, and the bound proteins were eluted with elution buffer at 25 °C for 30 min in the dark. The eluate was then combined with reaction buffer (1% SDC/100 mM Tris-HCl, pH 8.5/10 mM TCEP/40 mM CAA) and heated at 95 °C for 10 min to denature the proteins, reduce cysteines, and alkylate them. The eluates were diluted with an equal volume of water and subjected to overnight trypsin digestion at 37 °C, with trypsin added at a ratio of 1:50 (enzyme/protein, *w*/*w*). After digestion, the protein samples were purified using SDB desalting columns (SpeeAlly) and analyzed by LC-MS/MS (Thermo Scientific).

### 2.14. Histologic Assessment

Rat penile tissues were dissected, fixed overnight at 4 °C, and stored in 70% ethanol. The samples were then dehydrated and embedded in paraffin. After dewaxing and rehydration, five-micrometer sections were prepared for staining. Following blocking, immunohistochemical staining of the tissue sections was performed using anti-NEDD4 antibodies (Table S1). The slides were washed and incubated with an HRP-conjugated secondary antibody (Table S1). To assess tissue structure, Masson’s trichrome staining (G1006, Servicebio, Wuhan, China) was carried out according to the manufacturer’s guidelines. Additionally, the ratio of smooth muscle to collagen in the corpus cavernosum was quantified.

### 2.15. Statistical Analysis

The results are expressed as the mean ± standard deviation. For comparisons involving multiple groups, one-way ANOVA followed by Tukey’s post hoc test was applied. For comparisons between two variables, two-way ANOVA was utilized. All statistical analyses were conducted using GraphPad Prism 9, with a significance threshold set at *p* < 0.05.

## 3. Results

### 3.1. GPX4 Improves Cell Viability of CCSMCs Under High-Glucose Conditions

**Figure 1:**
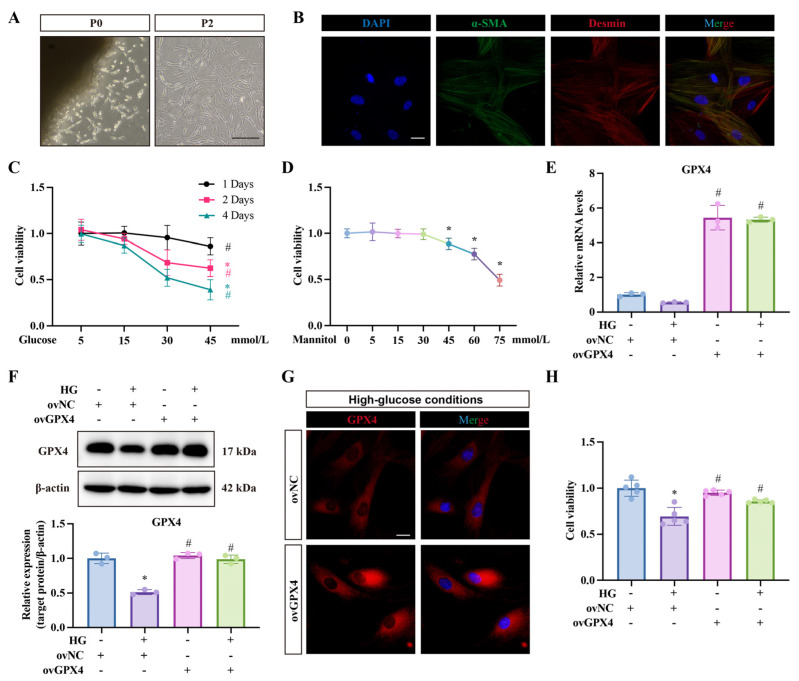
**Effects of ovGPX4 on cell viability under high-glucose conditions.** (**A**) Representative images showing cell morphology at passage 0 (P0) and passage 2 (P2). (**B**) Immunofluorescence staining of α-SMA and Desmin to confirm CCSMC identity. (**C**) Cell viability results after treatment with varying glucose concentrations and durations. (**D**) Cell viability results after treatment with different mannitol concentrations to control for osmotic pressure effects. (**E**) qPCR results showing GPX4 mRNA levels after the indicated treatments. (**F**) Representative Western blot analysis (upper) and semi-quantitative data (lower) for GPX4 protein after indicated treatments. Western blot original images can be found in Figure S1. (**G**) Immunofluorescence staining showing GPX4 expression after ovNC and ovGPX4 lentivirus transfection under high-glucose conditions. (**H**) Cell viability after ovGPX4 transfection. Scale bar: 50 μm. * *p* < 0.05 vs. control; # *p* < 0.05 vs. HG-treated group.

One week after rat corpus cavernosum tissue explants were cultured, cells began migrating out from the tissue (passage 0, P0). Following purification through differential adhesion and subsequent passaging (P2), the cells displayed a uniform, smooth muscle-like morphology (Figure 1A). We confirmed the identity of these purified primary CCSMCs by staining with α-SMA and Desmin, markers specific to smooth muscle cells, verifying that the majority of the cells were indeed CCSMCs (Figure 1B). To optimize the glucose concentration and exposure duration for this study, we tested various concentrations and time points, assessing cell viability using the CCK-8 assay (Figure 1C). The results showed a significant decrease in viability starting on day 2 at 30 mmol/L glucose. To rule out osmotic pressure effects from elevated glucose levels, we used mannitol as a control, finding that concentrations of 45 mmol/L or higher reduced cell viability (Figure 1D). Based on these results, we selected 30 mmol/L glucose for 2 days for further experiments. The results demonstrated that high glucose reduced GPX4 expression, which was restored upon ovGPX4 transfection (Figure 1E–G). Notably, following ovGPX4 lentivirus transfection, GPX4 mRNA levels in CCSMCs were significantly elevated compared to the control group, yet its protein expression only approximated normal levels. This discrepancy indicates the potential existence of a post-transcriptional feedback regulation mechanism in CCSMCs that maintains GPX4 protein expression near normal. Moreover, the Western blot results align with the observations from our previous animal experiments [20]. Additionally, GPX4 partially reversed the high-glucose-induced decline in cell viability (Figure 1H).

### 3.2. GPX4 Alleviates Ferroptosis in CCSMCs Under High-Glucose Conditions

**Figure 2:**
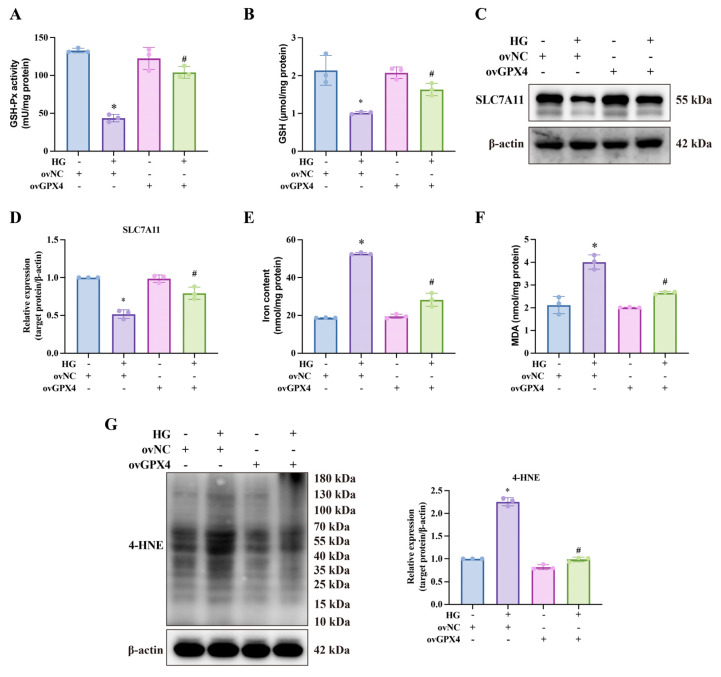
**Effects of ovGPX4 on anti-lipid peroxidation capacity and lipid peroxidation status under high-glucose conditions.** (**A**) Glutathione peroxidase (GSH-Px) activity and (**B**) GSH levels in CCSMCs under different treatments by GSH-Px or GSH assay kits. (**C**) Representative Western blot analysis and (**D**) semi-quantitative data for SLC7A11 protein levels after indicated treatments. (**E**) Total intracellular iron content and (**F**) malondialdehyde (MDA) levels in CCSMCs under different treatments. (**G**) Representative Western blot analysis (**left**) and semi-quantitative data (**right**) of 4-HNE protein levels after indicated treatments. * *p* < 0.05 vs. control; # *p* < 0.05 vs. HG-treated group.

**Figure 3:**
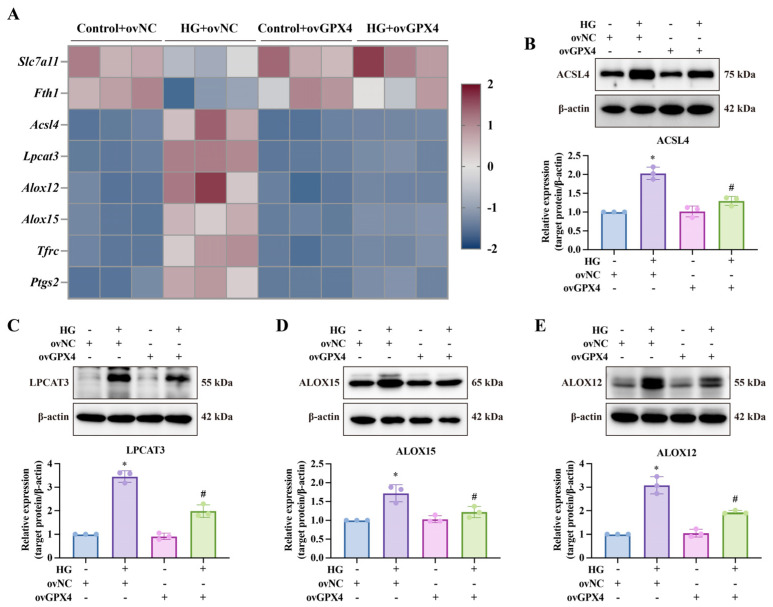
**Effects of ovGPX4 on ferroptosis-related molecules under high-glucose conditions.** (**A**) Heatmap displaying relative expression of ferroptosis-related genes detected by qPCR. (**B**–**E**) Representative Western blot analysis (upper) and semi-quantitative data (lower) for ACSL4, LPCAT3, ALOX15, and ALOX12 expression in CCSMCs. * *p* < 0.05 vs. control; # *p* < 0.05 vs. HG-treated group.

GPX4, a critical regulator of ferroptosis, uses GSH as a cofactor, acting as a phospholipid hydroperoxidase to mitigate lipid peroxidation. To explore the link between ferroptosis and DMED, we examined antioxidant levels and lipid peroxidation in CCSMCs carrying ovGPX4 lentivirus following high-glucose treatment. High glucose reduced GSH-Px activity and GSH levels, indicating a diminished capacity to resist lipid oxidation (Figure 2A,B). The partial restoration of GSH-Px activity by ovGPX4 implies that other GPX enzymes may also be impaired in CCSMCs under high-glucose conditions, hinting at a broader vulnerability in the antioxidative defense system [33]. To further explore how high glucose impacts GSH levels, we assessed the expression of SLC7A11, a key component of the cystine–glutamate anti-transporter (system Xc^−^) responsible for increasing intracellular GSH. High glucose reduced SLC7A11 expression, whereas ovGPX4 partially restored it (Figure 2C,D), mirroring the trends seen in GSH levels. Concurrently, MDA and 4-HNE levels, markers of lipid peroxidation, increased (Figure 2F,G). High glucose also raised intracellular iron levels, a key driver of ferroptosis (Figure 2E). Subsequently, we employed qPCR and Western blot to assess the expression levels of several key ferroptosis-related molecules (Figure 2C,D and Figure 3). Under high-glucose conditions, the expression of *Slc7a11* (responsible for cystine transport and GSH synthesis) and *Fth1* (encoding the ferritin heavy chain) was significantly reduced, both of which are critical for mitigating oxidative damage and maintaining iron homeostasis. Concurrently, the ACSL4-LPCAT3-LOXs pathway (ALOX15 and ALOX12) was activated, facilitating ferroptosis by integrating unsaturated fatty acids into phospholipid membranes. Moreover, the mRNA levels of *Ptgs2* (a marker of ferroptosis) and *Tfrc* (a key protein for iron uptake) were markedly increased. Notably, ovGPX4 lentiviral transfection effectively mitigated these aberrant molecular changes in CCSMCs.

### 3.3. GPX4 Ameliorates Oxidative Stress in CCSMCs Under High-Glucose Conditions

**Figure 4:**
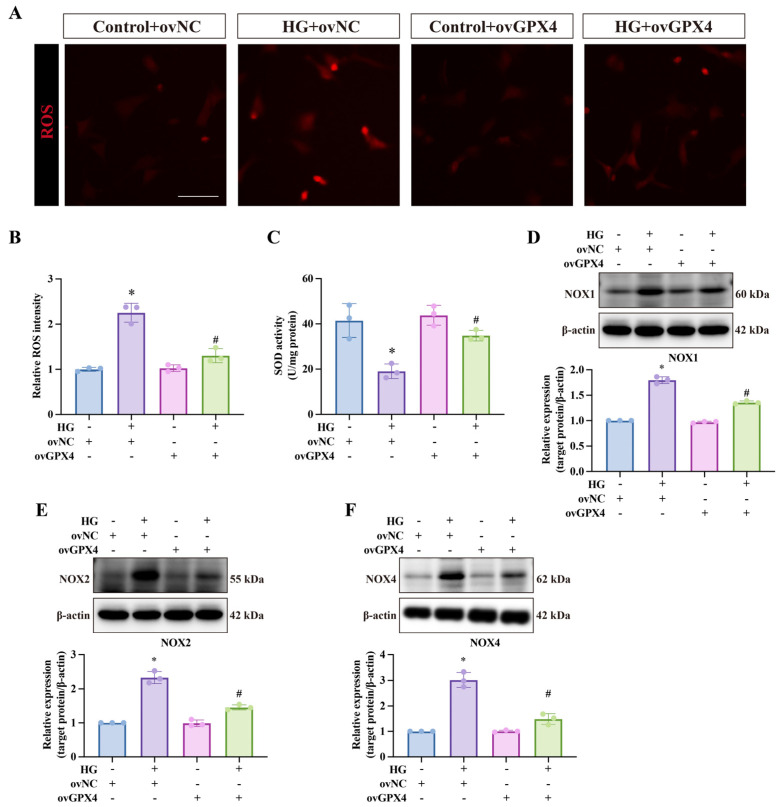
**Effects of ovGPX4 on oxidative stress under high-glucose conditions.** (**A**) Representative DHE staining images and (**B**) semi-quantitative analysis showing ROS levels across groups. (**C**) Superoxide dismutase (SOD) activity in different treatment groups. (**D**–**F**) Representative Western blot images (upper) and semi-quantitative analysis (lower) for NOX1, NOX2, and NOX4 expression in CCSMCs. Scale bar: 50 μm. * *p* < 0.05 vs. control; # *p* < 0.05 vs. HG-treated group.

Reactive oxygen species (ROS), downstream products of lipid peroxidation, are key drivers of ferroptosis. To explore the mechanisms of ferroptosis further, DHE staining was used to assess ROS levels. As shown in Figure 4A,B, the HG group exhibited the highest ROS levels, which were partially reversed by ovGPX4. Since superoxide dismutase (SOD), an antioxidant enzyme, scavenges ROS, we hypothesized that low SOD levels in a high-glucose environment may fail to counteract the detrimental effects of ROS on ferroptosis in CCSMCs. Indeed, SOD activity was lowest in the HG group, but ovGPX4 transfection significantly restored its levels (Figure 4C). Additionally, NADPH oxidases, including NOX1, NOX2, and NOX4, are major sources of intracellular ROS. All three were upregulated in CCSMCs under high-glucose conditions, and their levels were reduced following ovGPX4 transfection, mirroring the DHE staining findings (Figure 4D–F).

### 3.4. GPX4 Improves Smooth Muscle Function Under High-Glucose Conditions

**Figure 5:**
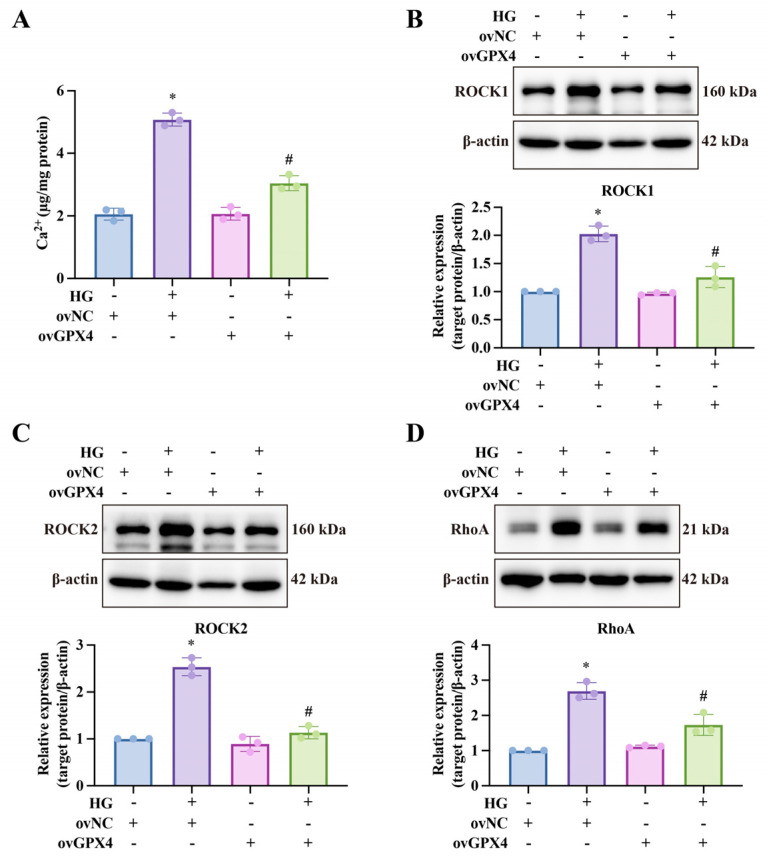
**Effects of ovGPX4 on smooth muscle function under high-glucose conditions.** (**A**) Calcium (Ca^2+^) levels across different treatment groups. (**B**–**D**) Representative Western blot images (upper) and semi-quantitative analysis (lower) of ROCK1, ROCK2, and RhoA expression in CCSMCs. * *p* < 0.05 vs. control; # *p* < 0.05 vs. HG-treated group.

Impaired relaxation of cavernosal smooth muscle is a key pathological mechanism in DMED. Previous studies have demonstrated that high glucose levels activate RhoA, which in turn stimulates its downstream targets, ROCK1 and ROCK2, resulting in elevated intracellular Ca^2+^ levels and increased Ca^2+^ sensitivity, thereby enhancing muscle contractility [16,17]. In our study, we first measured calcium levels, a critical factor in muscle contraction, and observed that the HG group showed the highest levels, whereas ovGPX4 significantly reduced them (Figure 5A). Similarly, high glucose upregulated RhoA, ROCK1, and ROCK2 expression, while ovGPX4 intervention effectively suppressed the activation of the RhoA/ROCK pathway (Figure 5B–D).

### 3.5. High Glucose Induces Proteolysis of GPX4 by Ubiquitin–Protease Pathway

**Figure 6:**
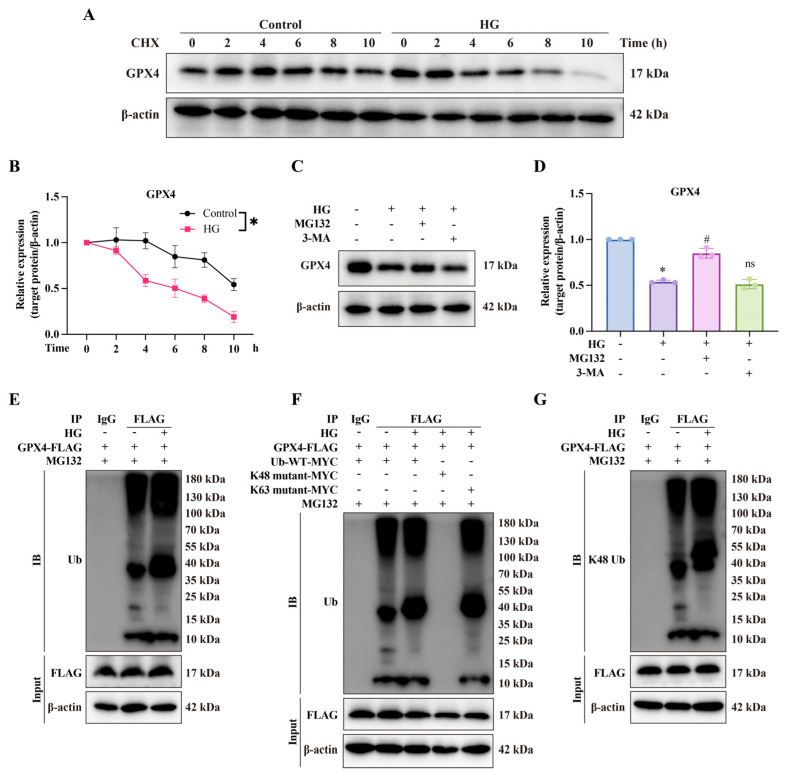
**High glucose induces GPX4 proteolysis via the ubiquitin–proteasome pathway.** (**A**) Representative Western blot images and (**B**) semi-quantitative analysis (**right**) of GPX4 expression with CHX treatment at indicated time points. (**C**,**D**) Western blot analysis shows that MG132 (10 μM) reversed HG-induced GPX4 degradation, whereas 3-Methyladenine (3-MA, 10 mM) did not, in CCSMCs. (**E**) Ubiquitination of GPX4 was detected by Co-IP in CCSMCs transfected with GPX4-FLAG plasmid. (**F**) Ubiquitination of GPX4 was further examined by Co-IP in HEK-293T cells co-transfected with GPX4-FLAG and MYC-tagged ubiquitin plasmids. (**G**) K48-specific ubiquitination of GPX4 was assessed by Co-IP in CCSMCs transfected with GPX4-FLAG plasmid. * *p* < 0.05 vs. control; # *p* < 0.05 vs. HG-treated group.

Under cellular stress, protein quality control is crucial for maintaining physiological homeostasis [34]. We hypothesized that GPX4 downregulation in a high-glucose environment might involve post-translational modifications. The in vitro degradation assay showed that GPX4 expression in CCSMCs gradually decreased with prolonged CHX treatment. Moreover, GPX4 protein levels declined more rapidly under high-glucose conditions, indicating that high glucose accelerates GPX4 protein degradation. (Figure 6A,B). This degradation was effectively inhibited by a 4 h treatment with the proteasome inhibitor MG132 but not by the lysosome inhibitor 3-MA (Figure 6C,D), indicating the involvement of the ubiquitin–proteasome system rather than the autophagy pathway. Polyubiquitination was also observed in GPX4-expressing cells (Figure 6E). To investigate further, lysine 48 (K48) and 63 (K63) point mutation studies showed that K48 polyubiquitin chains targeted GPX4 for proteasomal degradation (Figure 6F,G). Overall, these findings demonstrate that high glucose triggers GPX4 ubiquitination and subsequent degradation.

### 3.6. NEDD4 Mediates GPX4 Ubiquitination Degradation

**Figure 7:**
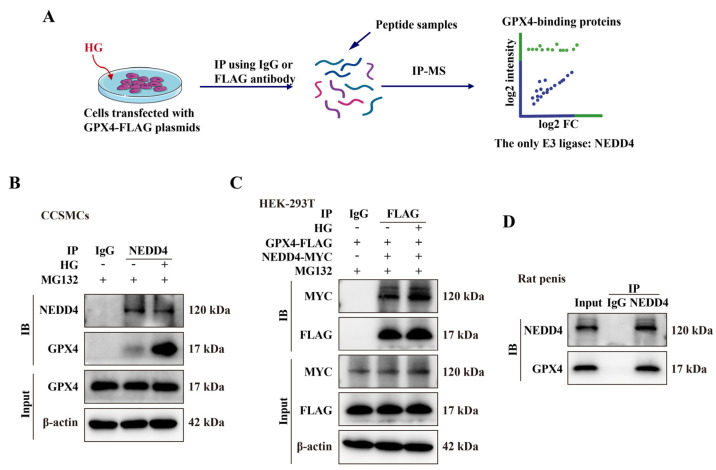
**Protein interaction between NEDD4 and GPX4.** (**A**) Schematic of the experimental design used to identify GPX4-binding proteins via IP-MS. (**B**) Co-IP verification of the interaction between GPX4 and NEDD4 in CCSMCs. (**C**) Co-IP verification of GPX4 and NEDD4 interaction in HEK-293T cells transfected with GPX4-FLAG and NEDD4-MYC plasmids. (**D**) Co-IP verification of GPX4 and NEDD4 interaction in rat penile tissue.

**Figure 8:**
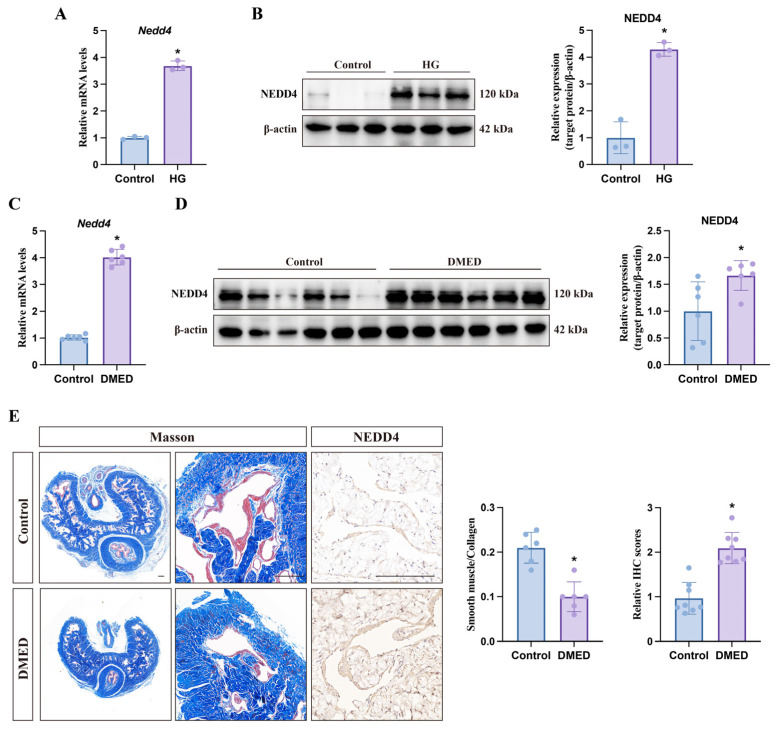
**Upregulation of NEDD4 in high-glucose-treated CCSMCs and DMED.** (**A**) qPCR analysis of NEDD4 expression following high-glucose treatment. (**B**) Representative Western blot images (**left**) and semi-quantitative analysis (**right**) of NEDD4 expression following high-glucose treatment. (**C**) qPCR analysis of NEDD4 expression in DMED rats. (**D**) Representative Western blot images (**left**), semi-quantitative analysis (**right**), and (**E**) immunohistochemical staining showing NEDD4 protein expression in DMED rats. Scale bar: 200 μm. * *p* < 0.05 vs. control group.

**Figure 9:**
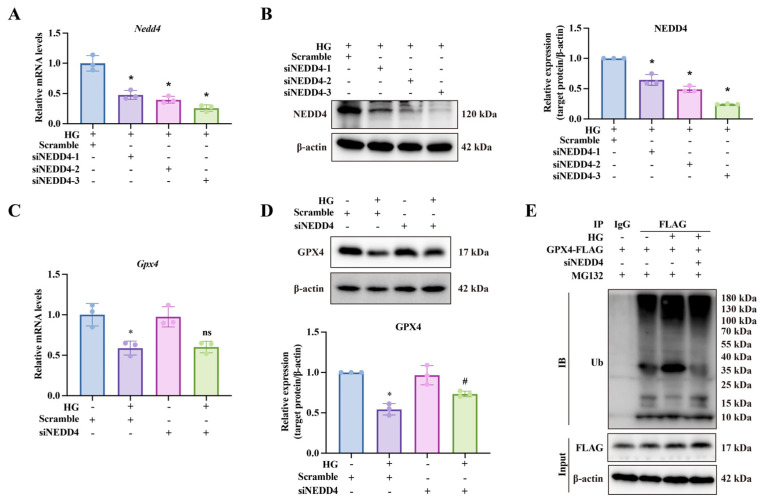
**NEDD4 mediates GPX4 ubiquitination and degradation.** (**A**) qPCR validation of NEDD4 siRNA efficacy. * *p* < 0.05 vs. HG group. (**B**) Western blot validation of NEDD4 siRNA efficacy. * *p* < 0.05 vs. HG group. (**C**) qPCR analysis of GPX4 expression after NEDD4 knockdown. * *p* < 0.05 vs. control; # *p* < 0.05 vs. HG-treated group. (**D**) Representative Western blot images (upper) and semi-quantitative analysis (lower) of GPX4 expression after NEDD4 knockdown. * *p* < 0.05 vs. control; # *p* < 0.05 vs. HG-treated group. (**E**) NEDD4’s role in GPX4 degradation was confirmed by Co-IP in CCSMCs transfected with NEDD4 siRNA.

To further investigate the molecular mechanism behind high-glucose-induced GPX4 ubiquitination, we performed IP-MS analysis to identify potential E3 ubiquitin ligases. Specifically, we transfected CCSMCs with the GPX4-FLAG plasmid and used an anti-FLAG antibody to enrich GPX4 and its interacting proteins. The peptide samples obtained were subsequently analyzed by IP-MS. The results indicated that NEDD4 emerged as the only significantly enriched E3 ligase interacting with GPX4-FLAG (Figure 7A). NEDD4 emerged as the only significantly enriched E3 ligase interacting with GPX4-FLAG (Figure 7A). This finding was validated through both endogenous and exogenous Co-IP, followed by Western blotting (Figure 7B–D). We then compared the mRNA levels of *Nedd4* in CCSMCs between the control group and the high-glucose group. qPCR results revealed a significant increase in *Nedd4* mRNA levels in the high-glucose group. This finding was further corroborated by Western blot analysis (Figure 8A,B). Additionally, we established a DMED rat model, with Masson’s staining confirming fibrosis as a common pathological feature in the penile tissue of DMED (Figure 8E). Subsequently, we examined NEDD4 expression in this model using qPCR, Western blot, and immunohistochemistry. The results revealed a significant upregulation of NEDD4 protein in the penile tissue of DMED rats (Figure 8C–E). To confirm NEDD4’s role in high-glucose-induced GPX4 ubiquitination, we conducted experiments with NEDD4 siRNA. siNEDD4-3 proved to be the most effective and was selected for further study (Figure 9A,B). NEDD4 knockdown did not affect GPX4 mRNA levels but significantly increased GPX4 protein expression under high-glucose conditions (Figure 9C,D). Additionally, Co-IP results showed that GPX4 ubiquitination was blocked in siNEDD4-transfected cells (Figure 9E). Collectively, these findings suggest that NEDD4 mediates GPX4 degradation in response to high glucose both in vitro and in vivo.

## 4. Discussion

Penile erection is a complex physiological process involving the coordination of nerves, endocrine cells, blood vessels, and other factors [35,36]. The structure and function of the corpus cavernosum are fundamental to this process, with CCSMCs playing a crucial role [37,38]. DM, a significant risk factor for ED, has been shown to reduce the number of CCSMCs, leading to impaired erectile function [39]. This reduction is often resistant to conventional medications like PDE5 inhibitors, contributing to poor treatment outcomes. To enhance therapeutic efficacy, it is essential to first identify the underlying causes of CCSMC loss and then develop strategies to improve cell viability.

GPX4 is an evolutionarily conserved enzyme that uses GSH as a cofactor, functioning as a phospholipid hydroperoxidase to reduce lipid peroxides to lipid alcohols [40]. This activity is crucial for maintaining cellular lipid homeostasis, preventing the accumulation of toxic lipid ROS, and thereby inhibiting ferroptosis [40]. Our previous research revealed that ferroptosis plays a role in the pathogenesis of DMED and that intracavernosal injection of GPX4 lentivirus significantly improves erectile function in diabetic rats, notably increasing the number and function of CCSMCs [20]. To explore whether GPX4-mediated regulation of ferroptosis directly affects CCSMC viability and function, this study involved culturing primary CCSMCs and transfecting them with GPX4 lentivirus. The findings demonstrated that GPX4 mitigated high-glucose-induced declines in cell viability, reduced lipid peroxidation, and decreased the expression of ferroptosis markers such as *Ptgs2* mRNA. However, since the characteristic morphological features of ferroptosis—such as shrunken, dense mitochondria—considered the gold standard for its identification [41], have not yet been confirmed, further studies employing transmission electron microscopy are needed to verify the presence of ferroptosis in CCSMCs under high-glucose conditions.

In studies of CCSMC models for DMED, there is inconsistency in the glucose concentrations and treatment durations used across different reports [9,32]. To provide a clearer reference for researchers and improve the reproducibility of experiments, this study systematically investigated the optimal glucose treatment conditions. We found that 30 mmol/L glucose treatment for 2 days effectively mimics the molecular changes induced by hyperglycemia in vivo, impacting CCSMC function without causing hyperosmolarity-related damage independent of glucose. However, a more precise concentration gradient between 30 and 45 mmol/L may yield improved results and requires further investigation. Additionally, the effects of varying high-glucose exposure durations on molecular cascades in CCSMCs remain unclear. It is also important to consider that DMED is influenced by factors beyond high glucose, such as advanced glycation end products, which future in vitro models should incorporate.

Recent reports have shown that hyperglycemia or high glucose induces oxidative stress, triggering ferroptosis in various organs and cell lines. In diabetic models, downregulation of the HIF-1α/HO-1 pathway leads to excessive ROS production, causing ferroptosis in renal tubules [42]. Similarly, HMGB1 regulates oxidative stress and ferroptosis via the Nrf2 pathway in mesangial cells under high-glucose conditions [43]. These studies highlight the critical role of oxidative stress in hyperglycemia- or high-glucose-induced ferroptosis. Our findings confirmed this, showing that GPX4 reduced ROS and 4-HNE (a lipid peroxidation marker) levels while increasing antioxidant species such as GSH and SOD. These results align with the known function of GPX4, which is essential for maintaining lipid homeostasis, preventing toxic ROS accumulation, and inhibiting ferroptosis.

The penis remains flaccid when the smooth muscle is contracted [44]. During sexual stimulation, an erection is triggered by the release of nitric oxide (NO) from non-adrenergic and non-cholinergic nerve fibers and acetylcholine from parasympathetic cholinergic nerves [3]. These signaling events lead to an increase in cyclic GMP (cGMP) levels, a reduction in intracellular Ca^2+^ concentrations, and smooth muscle relaxation. As the smooth muscle relaxes, blood fills the lacunar spaces within the corpora cavernosa, resulting in the erect state [45]. Notably, the RhoA/ROCK pathway plays a key role in regulating contraction and relaxation in CCSMCs [46]. RhoA, a small GTPase, activates ROCK, which phosphorylates the myosin-binding subunit of myosin light chain phosphatase (MLCP), inhibiting its function and promoting the Ca^2+^-dependent contractile response, keeping the penis in a flaccid state [15]. This study demonstrated that GPX4 reversed the elevated Ca^2+^ levels and the activation of the RhoA-ROCK1/ROCK2 pathway in CCSMCs induced by high glucose, indirectly confirming that GPX4 enhances the relaxation function of CCSMCs under high-glucose conditions.

**Figure 10:**
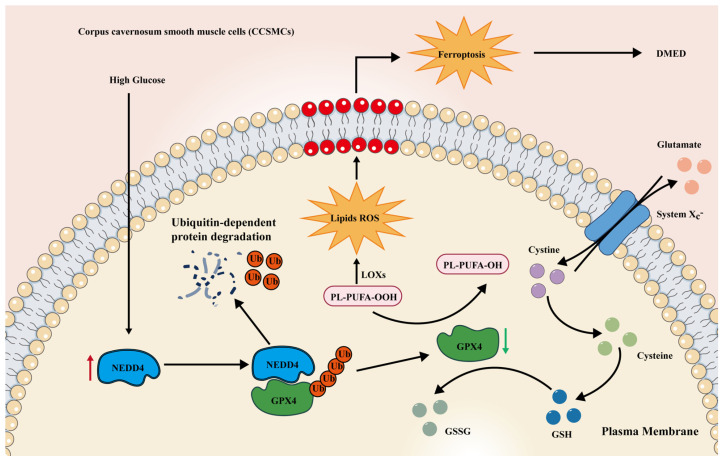
**Proposed mechanism of NEDD4-mediated ubiquitination of GPX4 in CCSMCs under high-glucose conditions.** The cystine–glutamate antiporter (system Xc^−^) transports cystine into the cell, where it contributes to the synthesis of glutathione (GSH). Under high-glucose conditions, an increased amount of polyunsaturated fatty acids (PUFAs) is catalyzed into lipid peroxides. High glucose also induces the expression of NEDD4, an E3 ubiquitin ligase that promotes GPX4 ubiquitination, leading to its degradation. As GPX4 levels decrease, there is less glutathione available to reduce lipid peroxides. This accumulation of lipid peroxides triggers ferroptosis in CCSMCs. The overall consequence of these molecular events is the development of diabetes mellitus-induced erectile dysfunction (DMED).

Proteostasis, the maintenance of a healthy proteome, is essential for cell metabolism, organelle biogenesis, stress adaptation, and the long-term viability of cells and organs [47]. Two primary degradation systems, the ubiquitin–proteasome system (UPS) and the autophagy–lysosome pathway (ALP), have evolved to manage these tasks. The UPS is the main route for degrading short-lived, misfolded, or damaged proteins, playing a crucial role in regulating cell signaling, transcription, and various cellular functions [48]. The ALP, on the other hand, removes large and potentially harmful cellular components, such as protein aggregates and dysfunctional or excess organelles, emerging as a vital adaptive mechanism [49]. Notably, these protein quality control systems are interconnected, interacting through multiple regulatory mechanisms. To investigate the downregulation of GPX4, we used inhibitors targeting the lysosome and proteasome pathways and found that GPX4 is primarily degraded through the UPS under high-glucose conditions. Further analysis revealed that the ubiquitination of GPX4 is mediated by NEDD4, consistent with recent studies showing NEDD4-dependent ubiquitination of DA quinone-modified GPX4 [50]. Considering that elevated NEDD4 levels in high-glucose conditions drive GPX4 degradation, we propose a model in which high glucose, iron, and ubiquitination work together to exacerbate ferroptosis in CCSMCs. This, in turn, contributes to the loss and dysfunction of CCSMCs, playing a role in the development of DMED (Figure 10).

However, several limitations in our study must be addressed. First, while NEDD4 has been identified as an interacting protein of GPX4, further investigation into the specific interaction mechanism is required. This includes identifying the interaction domains of both proteins using NEDD4 enzyme activity mutants, truncated forms, and GPX4 truncations, as well as determining whether the interaction is dependent on the E3 ubiquitin ligase function. Second, although NEDD4-mediated ubiquitination and degradation of GPX4 is one mechanism by which high glucose induces ferroptosis in CCSMCs, our findings, including a reduction in GPX4 mRNA, suggest that high glucose may also regulate GPX4 expression at the transcriptional level. Finally, while this study confirms the upregulation of NEDD4 expression both in vivo and in vitro, the exact role of NEDD4 in CCSMCs and DMED, along with the critical molecular changes in ferroptosis induced by its regulation, remains to be fully elucidated. Nevertheless, we have preliminarily shown that high-glucose stimulation of CCSMCs promotes NEDD4 expression, leading to the ubiquitination and degradation of GPX4, and thereby mediating ferroptosis.

## 5. Conclusions

In conclusion, GPX4 plays a critical role in protecting CCSMCs from high-glucose-induced ferroptosis and dysfunction. Its downregulation under diabetic conditions increases oxidative stress, contributing to ED. Restoring GPX4 expression improves cell viability and reduces oxidative damage. Additionally, NEDD4 modulates GPX4 stability via the ubiquitin–proteasome pathway, offering new insights into potential therapeutic strategies for DMED.

## Data Availability

The data presented in this study are available on request from the corresponding author.

## Supplementary Materials

The following supporting information can be downloaded at: https://www.mdpi.com/article/10.3390/biom14121552/s1, Table S1: antibodies used in this study; Table S2: list of siRNAs of NEDD4; Table S3: list of primers used in qPCR. Figure S1: original Western blot.
