# Supplementary material for: Neural Precursor Cell-Expressed Developmentally Downregulated Protein 4 (NEDD4)-Mediated Ubiquitination of Glutathione Peroxidase 4 (GPX4): A Key Pathway in High-Glucose-Induced Ferroptosis in Corpus Cavernosum Smooth Muscle Cells
Source: Biomolecules. 2024 Dec 5;14(12):1552. doi: 10.3390/biom14121552 (PMC11673994; doi:10.3390/biom14121552)

Figure 1S: original Western blot

Figure 1F GPX4

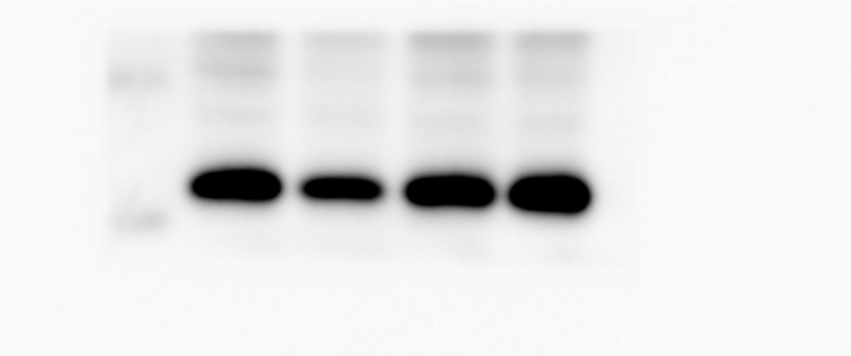

Figure 1F actin

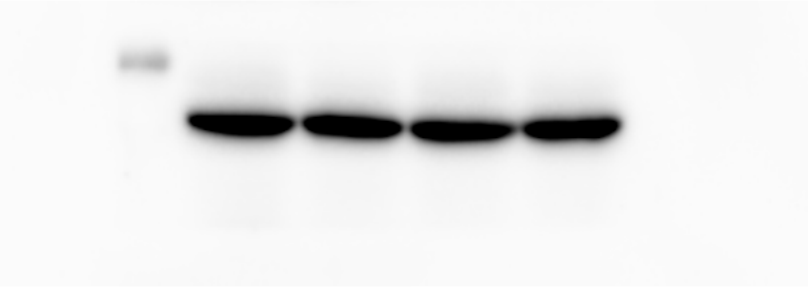

Figure 2C SLC7A11

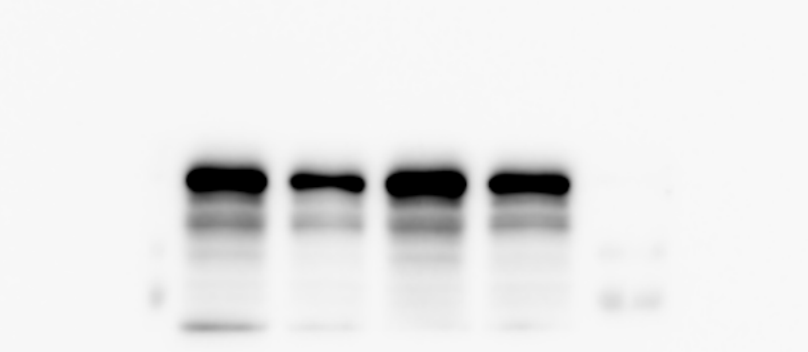

Figure 2C actin

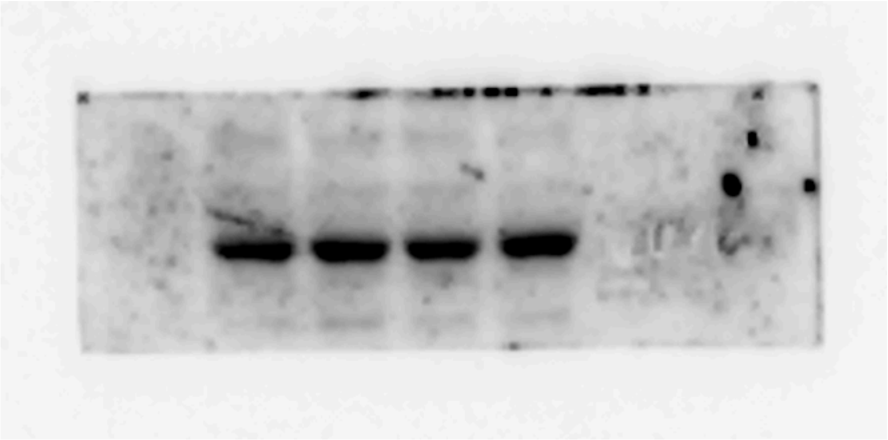

Figure 2G 4-HNE

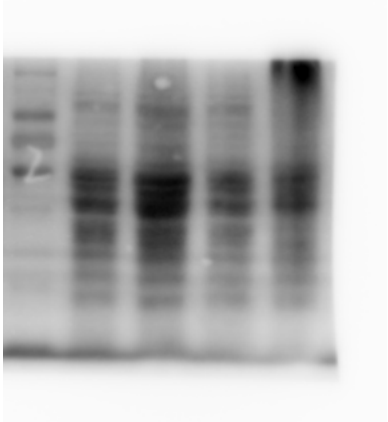

Figure 2G actin

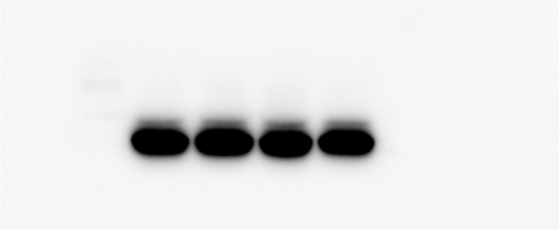

Figure 3B ACSL4

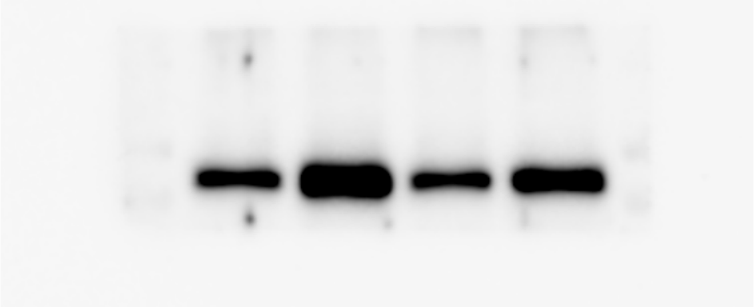

Figure 3B actin

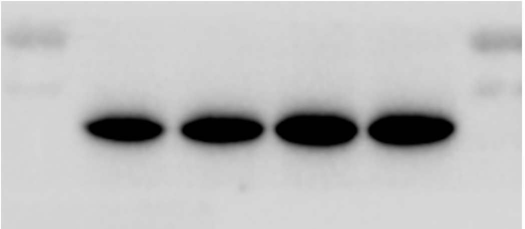

Figure 3C LPCAT3

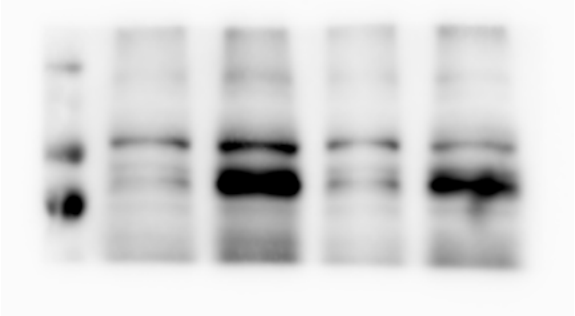

Figure 3C actin

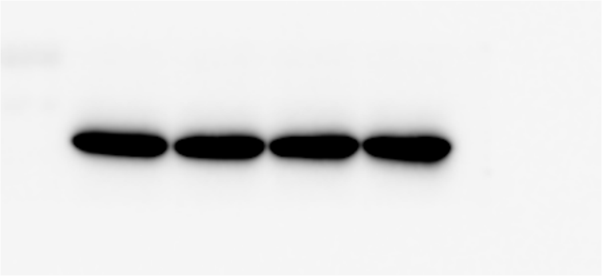

Figure 3D ALOX15

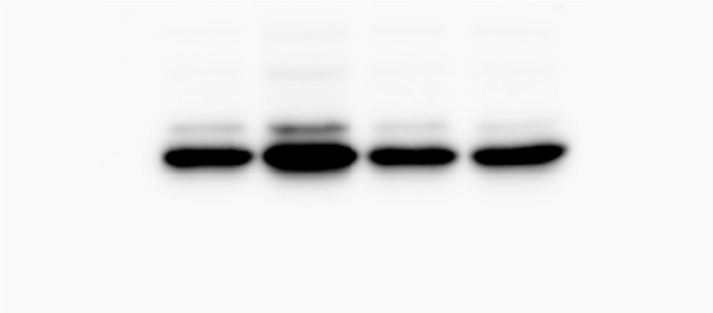

Figure 3D actin

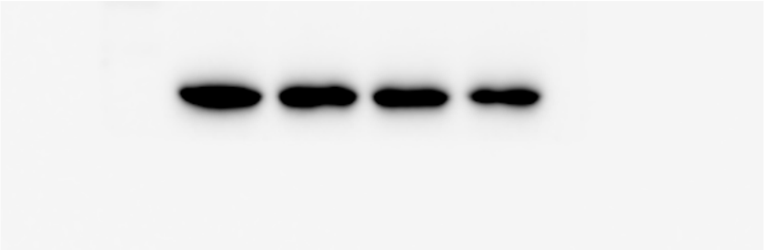

Figure 3E ALOX12

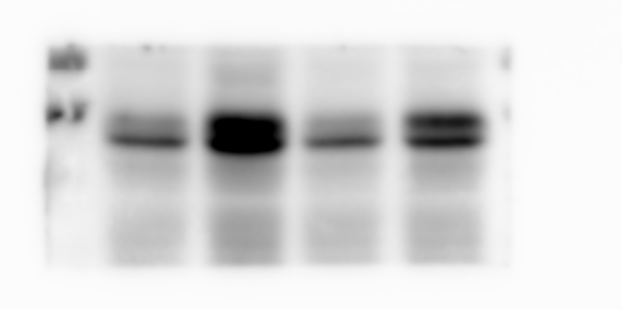

Figure 3E actin

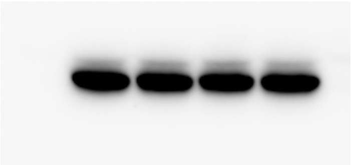

Figure 4D NOX1

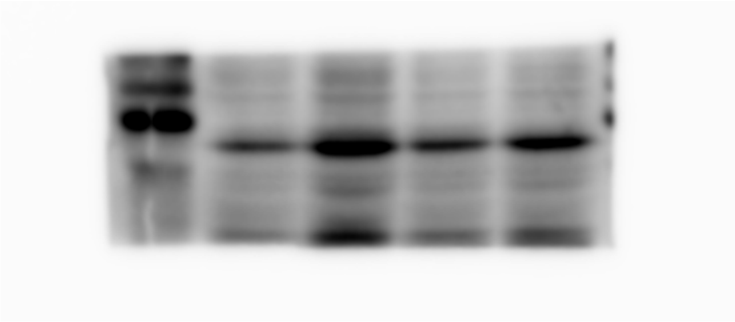

Figure 4D actin

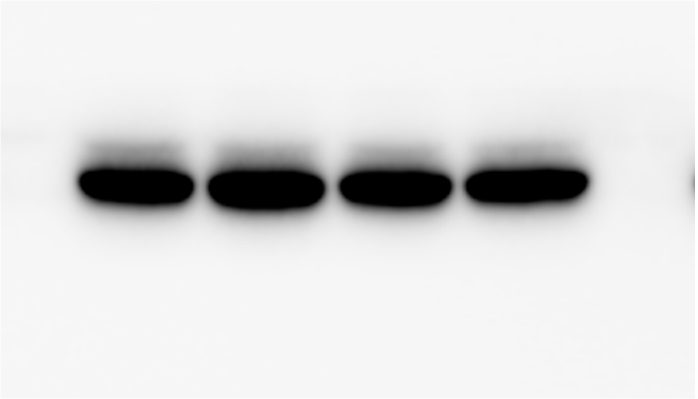

Figure 4E NOX2

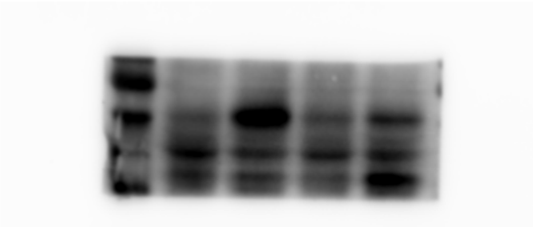

Figure 4E actin

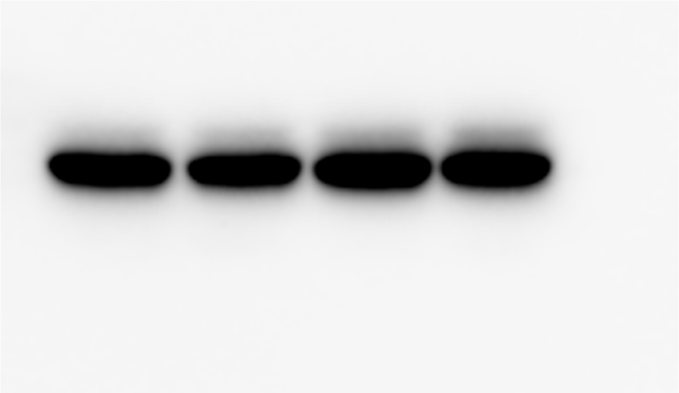

Figure 4F NOX4

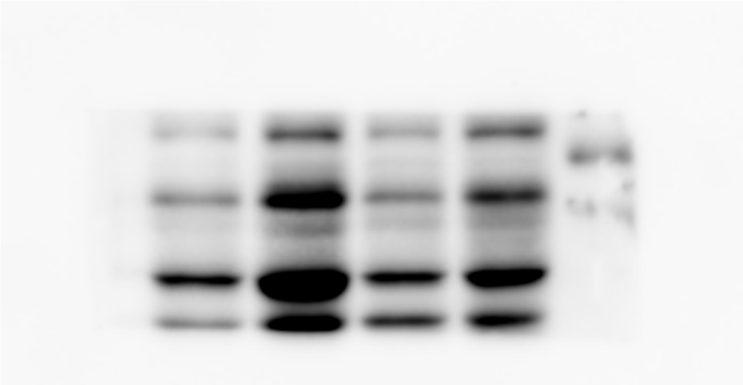

Figure 4F actin

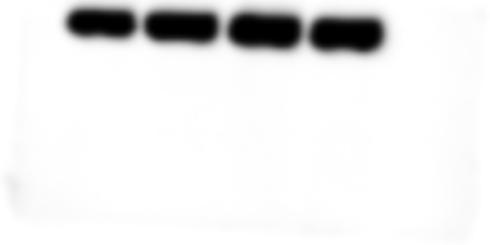

Figure 5B ROCK1

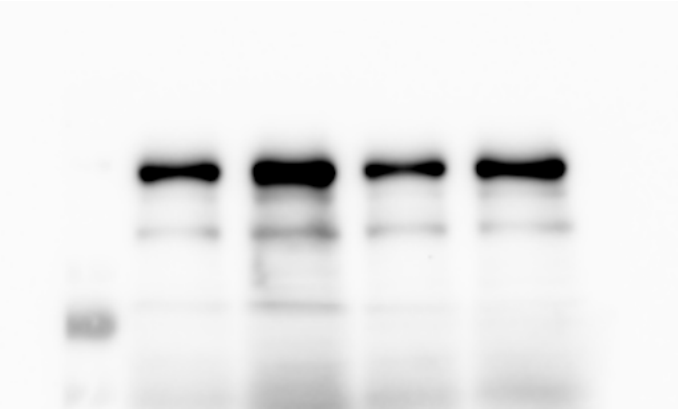

Figure 5B actin

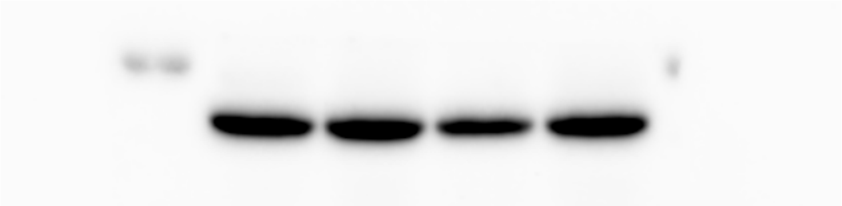

Figure 5C ROCK2

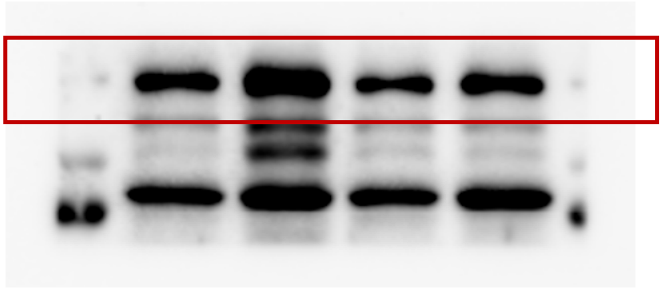

Figure 5C actin

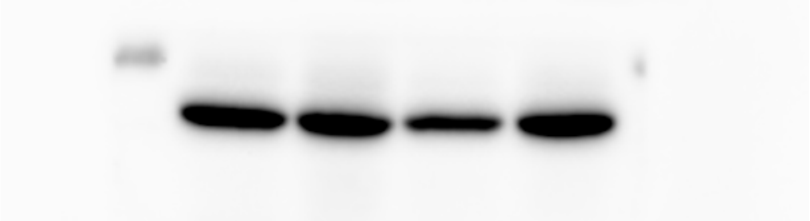

Figure 5D RhoA

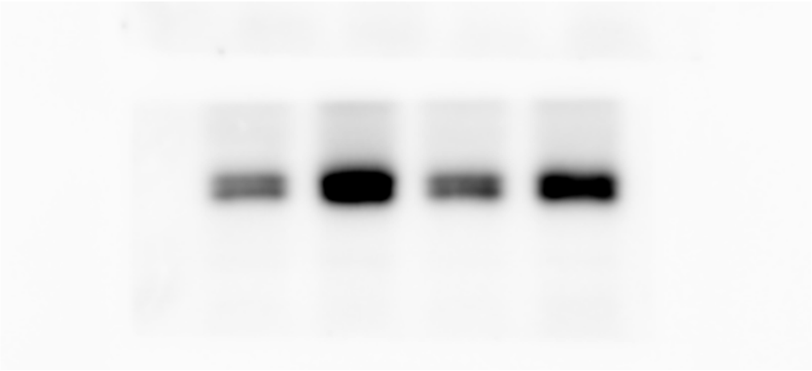

Figure 5D actin

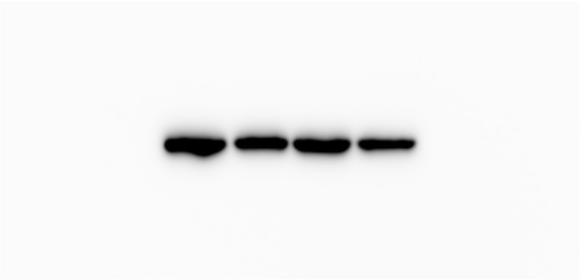

Figure 6A GPX4

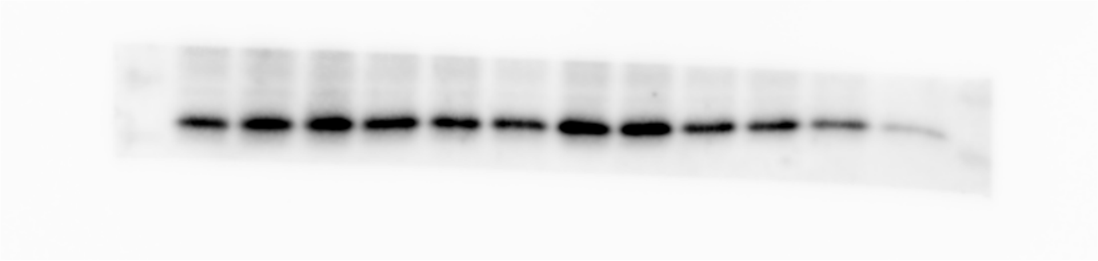

Figure 6A actin

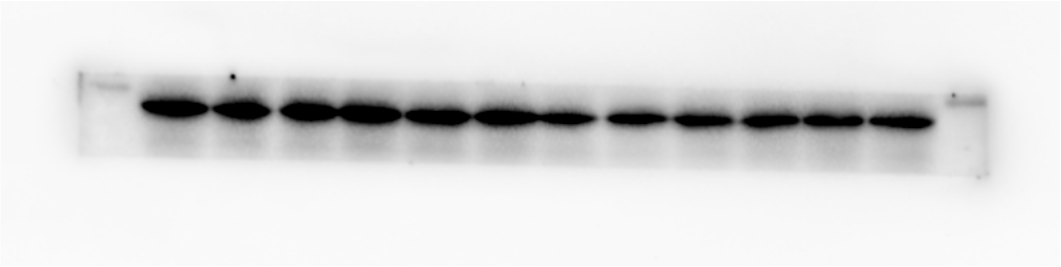

Figure 6C GPX4

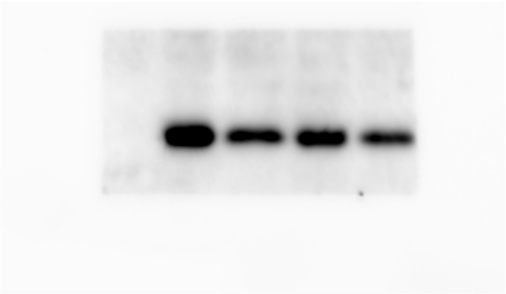

Figure 6C actin

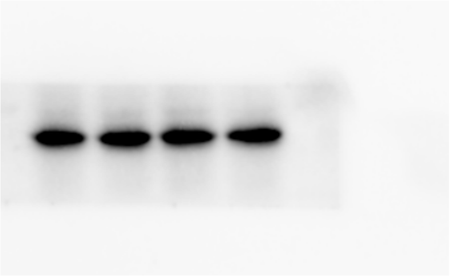

Figure 6E IB Ub

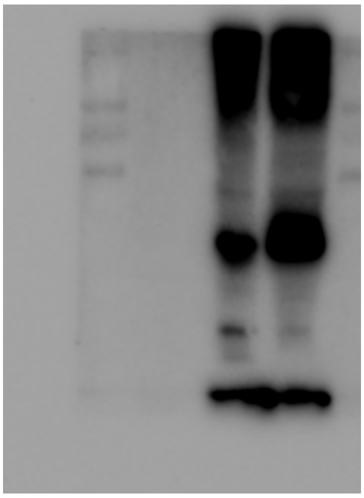

Figure 6E FLAG

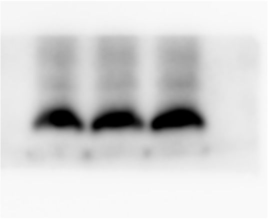

Figure 6E actin

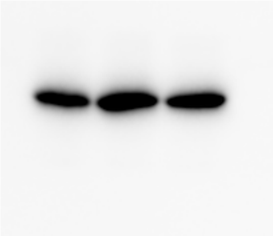

Figure 6F IB Ub

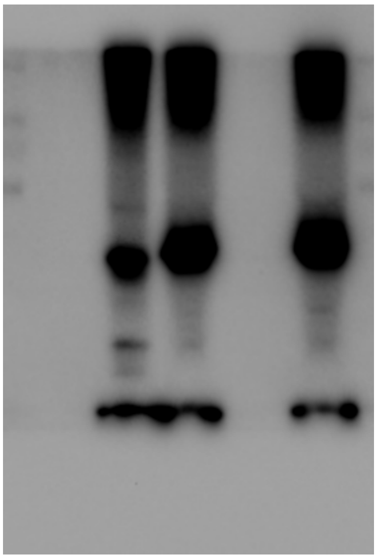

Figure 6F FLAG

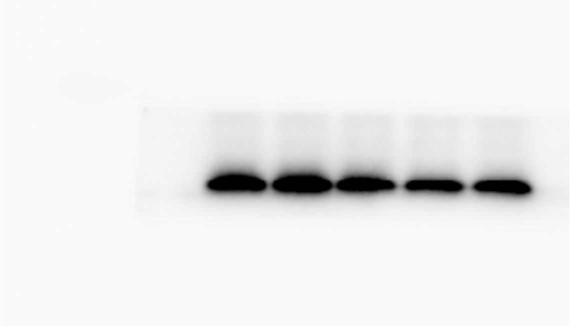

Figure 6F actin

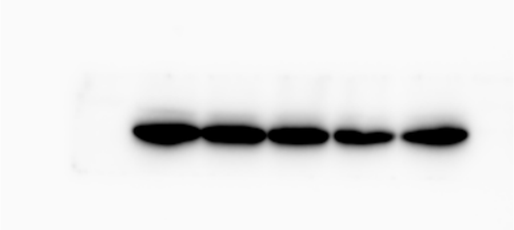

Figure 6G IB Ub

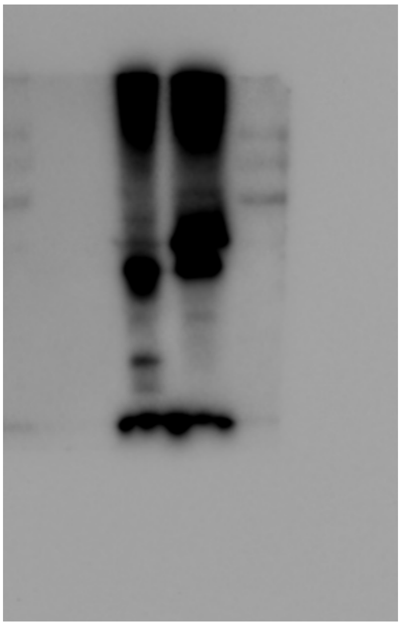

Figure 6G FLAG

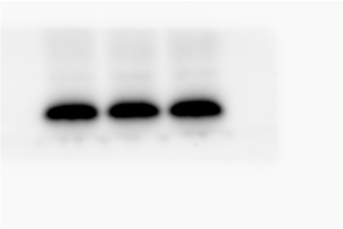

Figure 6G actin

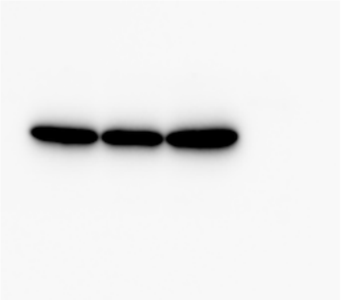

Figure 7B IB NEDD4

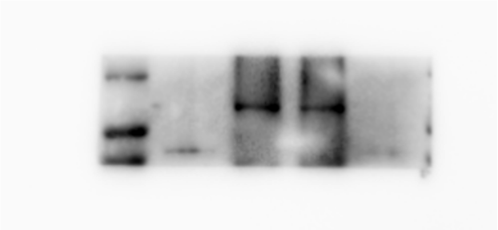

Figure 7B IB GPX4

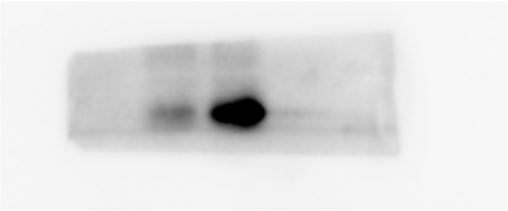

Figure 7B Input GPX4

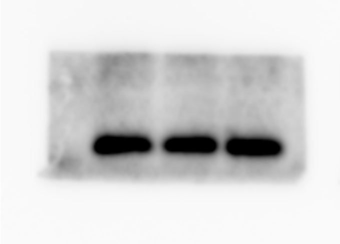

Figure 7B actin

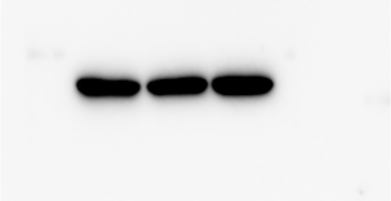

Figure 7C IB MYC

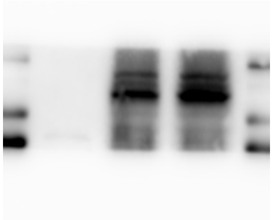

Figure 7C IB FLAG

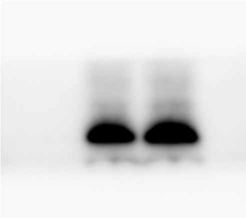

Figure 7C Input MYC

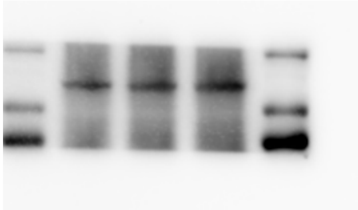

Figure 7C Input FLAG

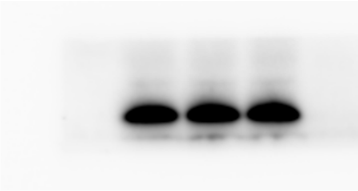

Figure 7C actin

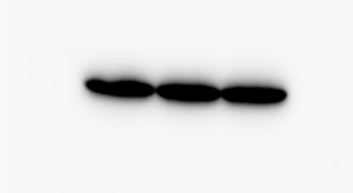

Figure 7D NEDD4

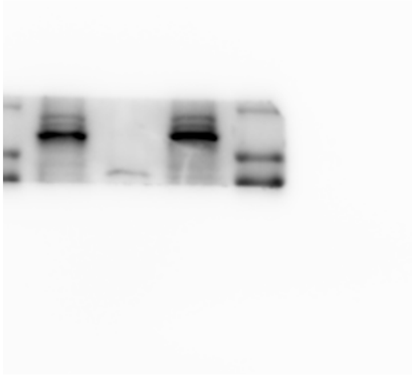

Figure 7D GPX4

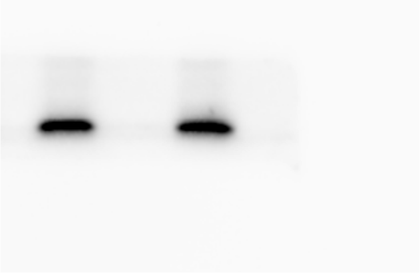

Figure 8B NEDD4

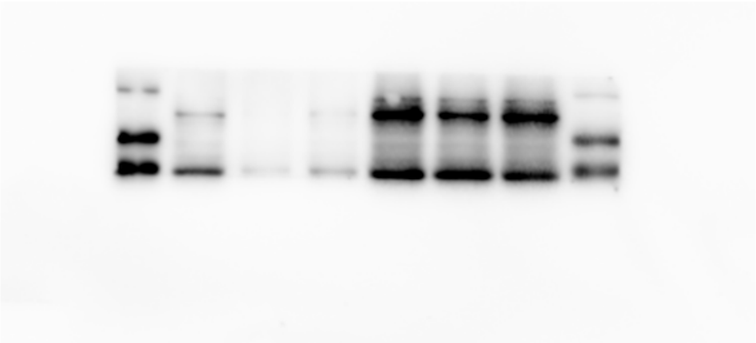

Figure 8B actin

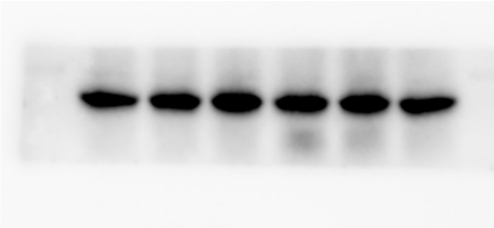

Figure 8D NEDD4

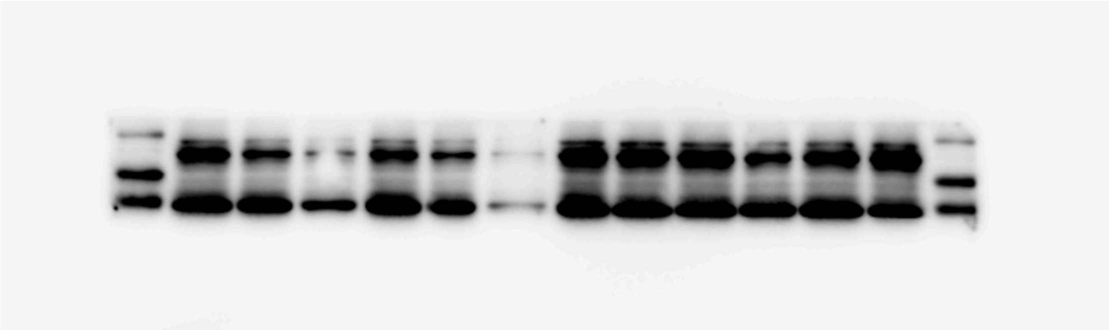

Figure 8D actin

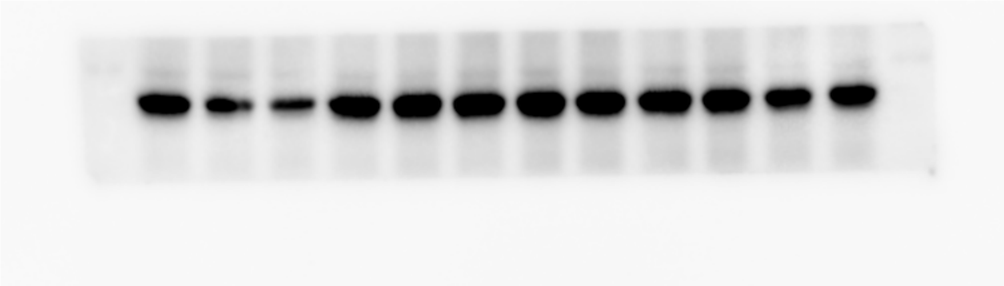

Figure 9B NEDD4

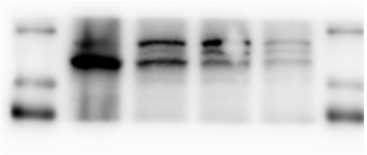

Figure 9B actin

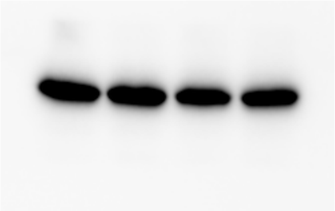

Figure 9D GPX4

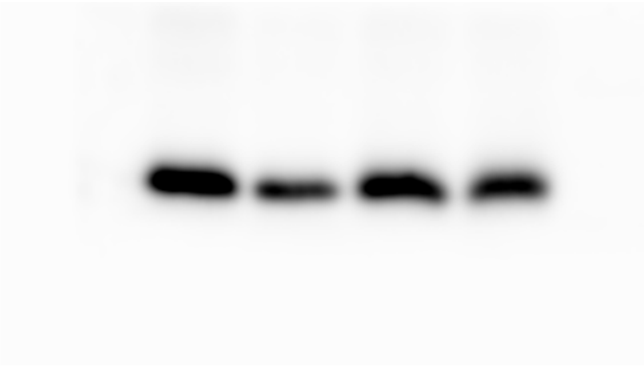

Figure 9D actin

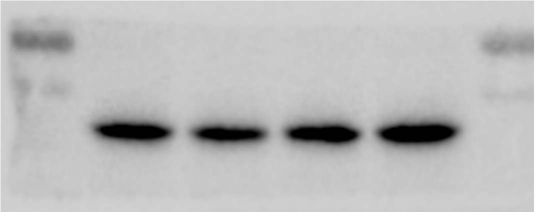

Figure 9E IB Ub

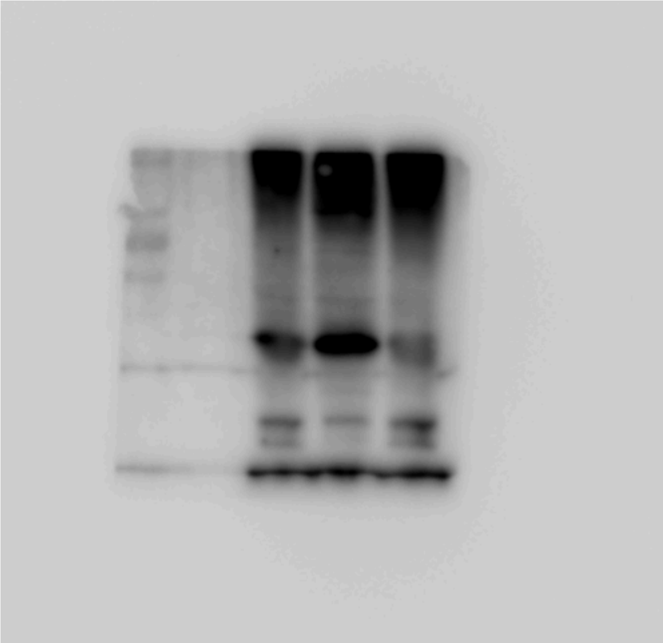

Figure 9E FLAG

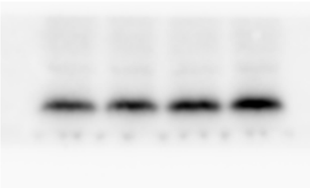

Figure 9E actin

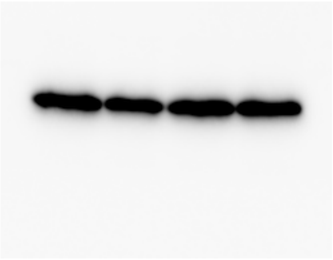

Supplement: Supplementary file 1 [file biomolecules-14-01552-s001.zip › Figure S1.pdf]
