# Supplementary material for: Neural Precursor Cell-Expressed Developmentally Downregulated Protein 4 (NEDD4)-Mediated Ubiquitination of Glutathione Peroxidase 4 (GPX4): A Key Pathway in High-Glucose-Induced Ferroptosis in Corpus Cavernosum Smooth Muscle Cells
Source: Biomolecules. 2024 Dec 5;14(12):1552. doi: 10.3390/biom14121552 (PMC11673994; doi:10.3390/biom14121552)
Supplement: Supplementary file 1 [file biomolecules-14-01552-s001.zip › Supplementary tables.pdf]

Table S1: antibodies used in this study.

Target	Manufacture (catalog number)	Source	Applications
GPX4	Boster (BM5231)	Rabbit	WB
GPX4	Affinity (DF6701)	Rabbit	IF
SLC7A11	Boster (BM5318)	Rabbit	WB
4-HNE	Abcam (ab46545)	Rabbit	WB
ACSL4	Affinity (DF12141)	Rabbit	WB
LPCAT3	Abclonal (A17604)	Rabbit	WB
ALOX12	Santa Cruz (sc-365194)	Mouse	WB
ALOX15	Abclonal (A6864)	Rabbit	WB
β-Actin	Abclonal (AC026)	Rabbit	WB
NOX1	Abcam (ab131088)	Rabbit	WB
NOX2	Proteintech (19013-1-AP)	Rabbit	WB
NOX4	Abcam (ab154244)	Rabbit	WB
α-SMA	Servicebio (GB111364)	Rabbit	IF
Desmin	Servicebio (GB12088)	Mouse	IF
ROCK1	Proteintech (21850-1-AP)	Rabbit	WB
ROCK2	Boster (PB0428)	Rabbit	WB
RhoA	Proteintech (10749-1-AP)	Rabbit	WB
Ubiquitin	Proteintech (10201-2-AP)	Rabbit	WB
Ub-K48	Abclonal (A3606)	Rabbit	WB
FLAG	Sigma (F1804)	Mouse	IP/WB
IgG	Santa Cruz (sc-2025)	Mouse	IP
NEDD4	Proteintech (21698-1-AP)	Rabbit	WB/IHC
IgG	Proteintech (30000-0-AP)	Rabbit	IP
MYC	Abclonal (AE070)	Rabbit	WB
HRP-conjugated Anti-Mouse IgG (H&L)	Abcam (ab6789)	Goat	WB
HRP-conjugated Anti-Rabbit IgG (H&L)	Boster (BA1054)	Goat	WB/IHC
HRP-conjugated Anti-Rabbit IgG (H)	Abclonal (AS122)	Mouse	WB for IB
HRP-conjugated Anti-Mouse IgG (H)	Abclonal (AS064)	Goat	WB for IB
Alexa Fluor 594-conjugated Anti-Mouse IgG (H&L)	Abcam (ab150116)	Goat	IF
Alexa Fluor 594-conjugated Anti-Rabbit IgG (H&L)	Abcam (ab150080)	Goat	IF
Alexa Fluor 488-conjugated Anti-Rabbit IgG (H&L)	Abcam (ab150077)	Goat	IF

Table S2: list of siRNAs of NEDD4.

siRNA No.	Target sequence
siNEDD4-1	CAGATGGAAGGGTCTTCTTCA
siNEDD4-2	GGAGCTGGACCTGAGATTTAT
siNEDD4-3	CAGATGGAAGGGTCTTCTTCA

Table S3: list of primers used in qPCR.

Species	Prime name	Sequence (5'→3')
Rat	GPX4-F	CATTCCCGAGCCTTTCAACCC
	GPX4-R	ATGCACACGCAACCCCTGT
Rat	SLC7A11-F	GGTTCAGACGATTGTCAGACAGAA
	SLC7A11-R	GGGCAGATGGCCAAGGATTT
Rat	FTH1-F	AACTACCACCAGGACTCGGA
	FTH1-R	TCATCACGGTCAGGTTTCTTT
Rat	ACSL4-F	TGGGCTGACAGAATCATGCG
	ACSL4-R	AACTGTATAACCACCTTCCTGC
Rat	LPCAT3-F	GCCTTAACAAGTTGGCGAC
	LPCAT3-R	CAAGAAGGTAGGCCATCTGGA
Rat	ALOX12-F	ACTTGACTTGGATCGCCTCC
	ALOX12-R	ATCAAACTCCTCCTCCTTGCC
Rat	ALOX15-F	GCAAGATGGGTGTCTACCGC
	ALOX15-R	CTTTGAATTCTGCTTCCGAGTCC
Rat	TFRC-F	CGGCCTATATGCTTGGGTAGG
	TFRC-R	TACAAGGGAGCACTCTGAAGC
Rat	PTGS2-F	CTCAGCCATGCAGCAAATCC
	PTGS2-R	GGGTGGGCTTCAGCAGTAAT
Rat	NEDD4-F	TTTCGGAGGACGAGGTATGG
	NEDD4-R	GCCAGACCTATGCCAGCTAT
Rat	ACTIN-F	ATCATTGCTCCTCCTGAGCG
	ACTIN-R	GAAAGGGTGTAAAACGCAGCTC
